# Assessing Different Types of HIV Communication and Sociocultural Factors on Perceived HIV Stigma and Testing among a National Sample of Youth and Young Adults

**DOI:** 10.3390/ijerph19021003

**Published:** 2022-01-17

**Authors:** Gamji M’Rabiu Abubakari, Martez D. R. Smith, Donte T. Boyd, S. Raquel Ramos, Courtney Johnson, Juan L. Benavides, Megan Threats, Junior L. Allen, Camille R. Quinn

**Affiliations:** 1 School of Public Health, Yale University, New Haven, CT 06510, USA; mohammed-rabiu.abubakari@yale.edu; 2 Center for Interdisciplinary Research on AIDS (CIRA), School of Public Health, Yale University, New Haven, CT 06510, USA; Raquel.ramos@yale.edu (S.R.R.); Megan.threats@rutgers.edu (M.T.); 3 School of Nursing, Rochester University, Rochester, NY 14642, USA; martez_smith@urmc.rochester.edu; 4 College of Social Work, The Ohio State University, Columbus, OH 43210, USA; benavides.35@buckeyemail.osu.edu (J.L.B.); Quinn.395@osu.edu (C.R.Q.); 5 School of Nursing, Yale University, Orange, CT 06477, USA; 6 Ryan Chelsea-Clinton Community Health Center, New York, NY 10036, USA; Courtney.johnson@ryancenter.org; 7 School of Communication and Information, Rutgers University, New Brunswick, NJ 08901, USA; 8 School of Social Work, Wayne State University, Detroit, MI 48202, USA; gm1306@wayne.edu; 9 Kirwan Institute for the Study of Race and Ethnicity, The Ohio State University, Columbus, OH 43201, USA

**Keywords:** US youth and young adults, HIV stigma, HIV testing, HIV communication, partner communication

## Abstract

In the United States, racial/ethnic and sexual youth and young adults (YYA) of color are disproportionately affected by HIV. Subsequently, YYA experience HIV stigma and engage in increased risk behaviors and reduced HIV testing. HIV communication has been identified as a potential buffer to HIV stigma, resulting in health-seeking behaviors, such as HIV testing. In this study, we respond to a meaningful gap in the literature by examining different types of HIV communication and their impact on HIV stigma and HIV testing in a diverse sample of YYA. We analyzed secondary data from the Kaiser Family Foundation National Survey of Teens and Young Adults on HIV/AIDS. A 40-question, web-based survey was conducted with 1437 youth (ages 15–24). Recruitment included a dual sampling method from households with: (1) listed phone numbers, (2) unlisted phone numbers, (3) telephones, (4) no telephone, and (5) only cell phone access. The purpose of the survey was to establish participants’ HIV knowledge, communication, experiences, and testing behaviors. Findings suggested an association between intimate-partner HIV communication, increased HIV testing, and reduced HIV stigma. We also identified differentials in HIV testing and stigma based on gender, income, age, and sexual minority status, explained by HIV communication. Further research is needed that examines ways to use intimate-partner HIV communication to reduce stigma and increase HIV testing among YYA of different sociodemographic characteristics and sexual orientations.

## 1. Introduction

To increase the identification of people living with HIV, reduce HIV infections, and improve linkages to care, reoccurring HIV testing for HIV key populations is recommended [1,2,3]. The Centers for Disease Control and Prevention (CDC) recommends HIV testing every 3 to 6 months for sexually active and high-risk youth and young adults (YYA). They also recommend incorporating HIV screening in the routine health care of adolescents in the United States [4]. However, YYA have lower HIV testing rates and are more likely than any other age group to be unaware of their HIV status [5]. Consequently, YYA are at severe risk for HIV transmission and disease progression [5,6,7,8,9]. In 2018, 21% of YYA comprised new HIV diagnoses, and close to 48% lived with HIV [4]. Yet only 55% of youth living with HIV know their status compared to 86% of the general population living with HIV [4].

The burden of HIV in YYA varies by gender, race, sexual orientation, and socioeconomic status, ages 15 to 24 years old [2,4,7,10]. The majority of new HIV diagnoses among women were attributed to heterosexual contact. Among women diagnosed with HIV in 2019, Black girls and women accounted for 61%, 16% were Latina, and 19% were White [11]. Among men, heterosexual contact contributed to 10% of HIV diagnoses in 2018; and Black heterosexual men are diagnosed at significantly higher rates than White heterosexual men. New HIV diagnoses remain high among Black (26%) and Latino (21%) MSM. About three out of four Black MSM diagnosed with an HIV infection occurred among young Black MSM, and about two out of three of Latino MSM diagnosed with an HIV infection occurred among young Latino MSM. This suggests an urgent need to focus on scaling up HIV testing and linkage to care among YYA, especially among the most affected demographics (Black and Latino MSM) [4]. HIV rates among young men were identified as higher due to the over-representation of gay, bisexual, and other men who have sex with men (MSM) (92%) when compared to heterosexual men (3%) [4]. Among racial differences in the US, Black youth were eight times more likely than White youth and two times more likely than Latino youth to contract HIV [10].

YYA are at an increased risk of HIV due to low condom use, lack of HIV knowledge, multiple sexual partners, and sex with older MSM [12,13] Because of this, it is critical for YYA and Black and Latino MSM to routinely check their HIV status. Stigma (internalized, perceived, and anticipated) has influenced YYA access and use of HIV testing [13,14,15,16,17]. Stigma refers to the occurrence of negative perceptions or associations of individual characteristics or phenomenon to rejection, unfair treatment, and/or discrimination [15,16,17,18,19]. Internalized stigma occurs when individuals attribute negative perceptions to their personal attributes and sometimes turn to undermine their self-value [15,16,17,18,19]. Perceived stigma occurs when one begins to question and build negative thoughts about what others may ascribe to them due to their stigmatized identities [15,16,17,18,19]. Anticipated stigma occurs when one expects to be stigmatized or rejected in a setting or by others. Young Black and Latino MSM often deal with these kinds of stigma within themselves, friends, immediate family members, and the community [13,15,16,17,18,19]. Stigma has been correlated with increased HIV transmission rates, risk behaviors, and poorer health outcomes. Experiences of stigma and racism have increased internalized and anticipated stigma and negatively correlate with a willingness to engage with HIV testing and utilization of HIV services [13,14,15]. Hence the efforts to increase HIV testing remain intrinsically connected to raising awareness and reducing HIV stigma among YYA, especially Black and Latino MSM and their communities.

Recommendations from previous studies note that to reduce HIV stigma, increase HIV testing, and reduce HIV transmission, YYA need to be subjected to increased HIV communication and education at all levels [20]. Sexual health communication is a strategy that can improve understanding of HIV transmission, risk behaviors, and prevention techniques [21,22]. This may result in increased HIV testing, early diagnoses, and linkages to care [20,23]. Sexual health communication is beneficial for young adults since they are at a crucial development period as they begin to develop and define their personal beliefs and values. Sexual health communication could be channeled through schools, health care settings, community youth centers, religious institutions, youth groups, and families [23]. Previous studies have shown that such communications can increase HIV testing and estimate that adolescents, including sexual minorities who receive HIV and sexual health communication, have more chances of testing for HIV than others who do not receive any communication about HIV [24,25].

The Current Study. Despite the relevance of HIV and sexual communication on HIV testing, little research has examined the relationship between sexual health communication and HIV testing and stigma among YYA, especially young Black and Latino MSM. This study examines the relationship between sexual health communication and HIV testing, and HIV stigma among YYA. This study also explores these relationships among the participants based on race, age, and sexual orientation.

## 2. Method

We analyzed secondary data from the Kaiser Family Foundation National Survey of Teens and Young Adults on HIV/AIDS. This survey assessed the knowledge, stigma, beliefs, and comfort of YYA around HIV. The 40-question, web-based survey was conducted with 1437 youths (aged 15–24) from 21 September 2012, through 1 October 2012. The survey respondents were members of the Knowledge Panel, a randomly drawn representative national panel of households selected using address-based sampling methods to participate by telephone. Knowledge Panel surveys use a dual sampling method that includes households with: (1) listed phone numbers, (2) unlisted phone numbers, (3) telephones, (4) no telephone, and (5) only cell phone access. The participants completed self-administered mail and web surveys, and households were provided with technology to access the internet, if necessary. This differs from other forms of internet research that include only individuals who can already access the internet.

Due to the sensitive subject matter, the parents of those participants aged 15–17 were provided a summary of the survey and had to consent for their children to participate. Of the total number of youths contacted, 77% of parents allowed their children to participate. The data were weighted to balance the sample demographics with estimates of the national population collected by the Census Bureau in August 2012. Since we analyzed publicly available secondary data, ethical approval was not required.

### Measures

The two outcome variables of this survey include HIV testing and HIV stigma. HIV testing, in this study, was based on the participants’ responses to the following: “Have you, yourself, ever been tested for HIV?” The responses were coded 0 for a negative response and 1 for an affirmative response. HIV stigma was measured using a 5 item, 4-point response scale (ranging from 1 = very comfortable to 4 = very uncomfortable), and sample questions consist of: “Working with someone who has HIV or AIDS”, “Having a roommate who is HIV-positive”, and “Having a close friendship with someone who is HIV-positive?” with lower scores indicating lower stigma. The Cronbach alpha is 0.90.

Several measures were used to assess HIV communication variables. Several contextual variables were collected. A single-item measured HIV communication, 4-point response scale (ranging from 1 = Never to 4 = Often) asked whether the respondents had held a conversation with someone about HIV/AIDS or other sexually transmitted infections (STIs) in the past year [7,10]. The overall mean for this item is 1.96 (*SD* = 0.91). *Glad the person brought it up* was measured by a single-item, 4-point response scale (ranging from 1 = Strongly disagree to 4 = Strongly agree) asked respondents, “If someone you were seeing romantically suggested that you get tested together for HIV, would you be glad the person brought it up?” A higher score indicated greater agreement. The overall mean for this item is 1.84 (*SD* = 0.82) [10].

The following independent variables are a series of single items, were evaluated on a 2-point response scale (ranging from 0 = *No, would not* to 1 = *Yes, would like more information*) and asked respondents the following questions: “Please tell me whether (or not) this is something you would like more information about: “How to talk to a partner about getting tested for STDs (including HIV)” (*M* = 1.35, *SD* = 0.47) and “How to talk to a partner about using condoms” (*M* = 1.27, *SD* = 0.44) [10].

Perceived HIV stigma in the US was measured using a single item, 4-point response scale (ranging from 1 = a lot to 4 = none at all) and asked respondents the following question: “How much stigma if any, do you think there is in the US today around HIV/AIDS”.

HIV knowledge was measured using a seven-item, 4-point response scale (ranging from 1 = strongly disagree to 4 = strongly agree). Sample questions consist of: “Unless you have sex with a lot of people, HIV is not something you have to worry about” and “HIV can only be spread when symptoms are present”. With higher scores indicating more significant HIV beliefs. Cronbach alpha is 0.90.

Condom use was measured using a single item, 4-point response scale (ranging from 1 = never to 4 = all of the time) and asked respondents the following question: “In your current or most recent sexual relationship, how often, if at all, do you use condoms?”.

Sociodemographic characteristics were also collected. Participants were asked to indicate their age, race and ethnicity, gender, sexual orientation, and household income. Age and household income are continuous variables. Gender was coded 0 if male and 1 if female. Race and ethnicity were coded as 1 = White, 2 = Black, 3 = Hispanic, 4 = Other, Non-Hispanic, and 5 = More than Two Races. Sexual orientation was coded as 1 = heterosexual, 2 = gay, 3 = lesbian, 4 = bisexual, and 5 = other.

## 3. Statistical Analysis

All analyses were conducted on observations that included non-missing data for the three outcomes: HIV testing, sexual health communication, and sexual risk behaviors. Statistical association tests were conducted between the measures described in the Method section and the three outcomes. Table 1 provides sample characteristics for the study sample. Table 2 provides a bivariate correlation analysis for all the continuous variables: HIV stigma, perceived stigma, HIV knowledge, HIV communication, bringing up the topic of HIV, HIV communication with partner, and age. Table 3 provides a chi-square test with categorical variables: household income, education attainment, sexual orientation, race and ethnicity, and gender by HIV testing. Table 4 presents a multivariate analysis regression model that examines the predictor variables: perceived stigma, HIV communication, bringing up the topic of HIV, HIV communication with a partner, HIV knowledge and condom use along with the accompanying covariates (gender, age, race, and ethnicity, household income, sexual orientation), and the outcome variable: HIV stigma. Table 5 presents a logistic regression analysis with predictors and covariates and the outcome variable, HIV testing. A mean score of the scale items was generated for participants with non-missing data for survey scales. All analyses were conducted using STATA 17, STATACorp LLC, College Station, TX, 77845-4512, USA.

### 3.1. Sample Characteristics

We analyzed data from 1437 participants (Table 1). Most of the participants self-identified as female (56%). The average age was 20 years (*SD* = 3.02). The majority of YYA self-identified as heterosexual (91%). Most of the sample (61%) was between 18 and 24 years of age and the average household income was between $35,000 and $39,000. Fifty-three percent reported being sexually active during the study period, and 75% said they had not received an HIV test. Approximately 50% reportedly that perceived stigma exists in the US. In addition, 67% of the sample reported stigmatizing views towards HIV-positive individuals; for instance, 35% stated that “Having an HIV-positive roommate” was uncomfortable. The majority of the sample had negative beliefs about HIV. Only 50% reported communicating about HIV in general, and 78% said it was difficult bringing up the topic of HIV to their partner. Approximately 40% of the sample never communicated about HIV due to HIV stigma.

### 3.2. Bivariate Results

Table 2 provides bivariate correlations between the continuous variables and the outcome variable of HIV stigma. A positive correlation also existed between HIV communication and HIV stigma (*r* = 0.16, *p* < 0.001). Bringing up the topic of HIV was statistically significant and negatively associated with HIV stigma for YYA (*r* = 0.21, *p* < 0.001).

Table 3 provides chi-square results comparing sample characteristics by HIV testing. A chi-square test of independence was performed to examine the relation between sexual orientation and HIV testing. The relation between these variables was significant, *X*^2^ (4, N = 1437) = 14.65, *p* = 0.001. A chi-square test of independence showed a significant association between race and ethnicity and HIV testing, *X*^2^ (2, N = 1437) = 109.105, *p* = 0.001.

### 3.3. Multivariate Results

As presented in Table 3, the overall model was statistically significant for HIV stigma. Bringing up the topic of HIV with a partner was statistically significant and negatively associated with HIV stigma (β = −0.13, *p* < 0.001). Individuals who wanted information about how to communicate HIV with a partner were positively associated with HIV stigma (β = 0.10, *p* = 0.004). HIV knowledge was negatively associated with HIV stigma (β = −0.24, *p* < 0.001). Age was statistically significant and positively associated with HIV stigma (β = 0.04, *p* < 0.001). Higher household incomes were statistically significant and positively associated with HIV stigma (β = 0.01, *p* = 0.014). Among sexual minorities, those who reported being gay (β = 0.82, *p* < 0.001) and bisexual (β = 0.46, *p* < 0.001) were both positively associated with HIV stigma.

As presented in Table 4, the overall model was statistically significant for HIV testing. Youth who engaged in communication about HIV were more likely to get tested for HIV than those who never communicated about HIV (OR: 1.68, 95%CI: 1.35, 2.09). Those who wanted to bring up the topic of HIV with a partner were less likely to get tested for HIV (OR: 0.79, 95%CI: 0.62, 1.00). Participants who wanted more information about communicating about HIV with their partner were more likely to get tested for HIV than individuals who did not want information (OR: 1.43, 95%CI: 1.16, 1.76). African American (OR: 2.32, 95%CI: 1.43, 3.77), Hispanic (OR: 1.62, 95%CI: 1.04, 2.53), and youth who reported multiple racial/ethnic identities (OR = 2.73, 95%CI: 7.19) were more likely to get tested for HIV than whites. Recent condom use was statistically significant and positively associated with HIV testing (β = 0.03, *p* < 0.001). Females were more likely to get tested for HIV than males (OR: 1.49; 95%CI: 1.03, 2.17). Participants who reporter lower household incomes were more likely to get tested for HIV than individuals who lived in households with higher incomes (OR:0.94, 95%CI: 0.90, 0.97).

## 4. Discussion

This study aimed to examine the relationship between different types of HIV communication on HIV testing and HIV stigma among YYA. We also examined these relationships based on race, income, age, and sexual orientation. We identified significant associations between HIV communication and HIV stigma, HIV communication, and other contextual factors on HIV testing. Several studies have highlighted the impact of stigma on HIV testing and linkage to care and the utility of sexual health communication in buffering HIV stigma and increasing HIV testing and linkage to care [18,26,27,28,29]. Our study provided significant insights about partner sexual health communication and HIV stigma and testing among YYA, necessary for intervention delivery that targets most at-risk populations in HIV science.

### 4.1. HIV Communication and HIV Stigma

Our findings highlighted the importance of sexual health communication in cultivating positive attitudes towards HIV and reducing HIV stigma among young adults. We found that young adults can lower their perceived HIV stigma when discussing HIV with their partners. YYA who were glad that their partner brought up the topic of HIV were less likely to have stigmatizing views towards HIV. This is consistent with other literature that showed the influence of YYA wanting to discuss HIV with their partner, impacting HIV prevention behaviors [30]. This is a significant contribution, as we must find ways to engage YYA and their partners around HIV to really understand their relationship dynamics and how these dynamics influence HIV stigma. We also must further try to understand the different types of discussion that are being had within these relationships.

Additionally, our findings suggested that increased knowledge about HIV in YYA was correlated to a lower HIV stigma. Although our finding on partner communication and HIV stigma among young adults fills a significant gap in understanding HIV communication and stigma, previous studies have illuminated the significance of communication in reducing the enacted and perceived stigma among HIV key populations [29,30,31]. HIV stigma has been identified as one of the most significant concerns that impede HIV conversation and improve HIV health outcomes among key populations [26,27]. Our study findings contribute to the literature in this area by identifying the need for heightened interventions that address stigma via partner communications among young adults. Our results are also concordant with previous studies and suggest that partner communication can increase knowledge on HIV and, by extension, reduce HIV stigma. [29,31].

Findings from this study are concordant with many studies on the relationship between identifying with a sexual minority status and experience with HIV stigma [32,33]. Despite a relationship between sexual health communication and reduced perceived HIV stigma, YYA who identify as gay or bisexual correlate with higher perceived HIV stigma. This has implications for HIV prevention and care among key populations such as young adults, especially MSM, who disproportionately carry the burden of HIV infections [28]. HIV stigma has a negative consequence for other key HIV prevention and treatment outcomes such as condom use, testing, linkage, and adherence to care [26].

### 4.2. HIV Communication and HIV Testing

Communication about HIV has been identified as a critical promoter of HIV testing and diagnoses and subsequent linkage to care [34,35,36]. Limited research showed that young adults who engage with HIV communication in schools or with families, providers, or peers have positive attitudes towards HIV testing compared to those who do not engage in any form of HIV conversations [34,35,36]. Our findings further this dialogue to call for the need for increased interventions that encourage HIV communication among young adults and their partners [34,35,36]. We found that both young adults interested in holding HIV communication and those who have such conversations have a greater chance of HIV testing than those who do not have plans to hold such discussions with their partners.

### 4.3. HIV Communication as a Critical Determinant of HIV Testing among Young Adults of Different Socioeconomic Statuses

Our findings illustrate the disparities in HIV testing alongside race, income, and gender among young adults. Our key finding suggested a negative relationship between HIV testing and White young adults compared to positive relations between Black and Hispanic young adults and HIV testing in our sample. This finding departs from a longstanding discourse on access to HIV testing and linkage to care where racial minorities are predominantly associated with lower access and use of HIV testing services in the United States [37,38,39]. This finding does not dismiss the existence of the long-established knowledge on low HIV testing among racial and ethnic minorities; it only accentuates the importance of HIV communication between partners in improving HIV testing [34,35,36]. In our sample, higher proportions of young adults who identify as White reported less communication about HIV than those who identify as Black or Hispanic, thus explaining why White young adults endorsed lower HIV testing. This significant finding shows that HIV scientists and policymakers could scale up HIV testing among key populations irrespective of the racial or ethnic identity of the target community if we channel resources into HIV communication strategies, especially between partners among young adults in the country.

Another unconventional finding in our study is that young adults who reported lower household incomes were more likely to get tested for HIV than individuals who lived in households with higher incomes. Scholars who examine social vulnerabilities rank lower income, together with racial minority status as factors associated with lower engagement with care [33,34,35,36,37,38,39,40]. Studies about HIV have found that lower income factors impede access to HIV services, including HIV testing [33,34,35,36,37,38,39,40]. Similar to our findings on racial disparities in HIV testing, HIV communication may explain why young adults with lower income in our sample were positively correlated with HIV testing compared to higher incomes. We had a higher representation of YYA who had engaged in HIV communication among lower-income groups compared to higher-income groups, this reechoing our call on focusing on increasing HIV communication among young adults since it has the potential to increase HIV testing and linkage to care [36]. Indeed, partner communication has been a critical determinant of HIV testing among young adults [36]. Not surprisingly, we identified higher HIV testing among young females than male participants, which corresponds with findings from previous studies. We surmise this may be due to females generally choosing to use health care services more than males due to reproductive health concerns and a higher likelihood of visiting providers than males [36].

## 5. Limitations

There were several limitations to this study. First, the data were cross-sectional, so we cannot account for longitudinal effects over time or temporal ordering. Second, our results may not be generalizable to the greater community of YYA of diverse geographic and genetic backgrounds. However, generalizability may be increased due to using a nationally representative sample. Additionally, it is important to note that this study overrepresented heterosexual YYA compared to MSM. Whereas we make some analysis to show the differences between our sample based on sexual orientation, we recognize that the sample of MSM remains small in the population, hence, limiting our ability apply our findings to the general MSM community. Our findings, however, could augment others to provide a broader understanding on HIV stigma, testing and community among YYA and among YYA who are MSM. Lastly, our study did not include anyone who self-identified as HIV positive in our results, which may impact our findings.

## 6. Future Research

Future research should investigate what types of discussions are happening between youths and their friends, sexual partners, and families. In gaining this understanding, we can learn if the same or different type of conversation is happening among these different types of networks, and what information is being provided about HIV in these different contexts. This will allow researchers and practitioners to target the information and find ways to correct it and is critical because sexual health communication can play an essential role in reducing HIV-related disparities and stigma, which is central to the HIV epidemic. Having sexual health communication between families, friends, and sexual partners can increase their comfort with talking about HIV and removing any fears about the disease.

## 7. Conclusions

This paper examined the role of sexual health communication about HIV stigma and HIV testing. We found that different types of communication about HIV can be complex but can reduce stigma and increase HIV testing among the most vulnerable populations. This is significant because improving communication about HIV among youths, their partners, and family can potentially reduce HIV stigma and increase HIV testing significantly. More research is needed on different types of communication about sex and HIV and how they influence intersectional stigma. Sexual health communication is a promising strategy to mitigate HIV risk and increase HIV testing in ethnic/racial YYA.

## Figures and Tables

**Table 1:** Sample Characteristics (N = 1437).

Variables	Frequency	Percentage
Race and Ethnicity		
African American or Black	271	39
Hispanic	322	46
Other, Non-Hispanic	57	8.0
Two + Races, Non-Hispanic	51	7.2
Gender		
Male	312	44
Female	392	57
Sexual Orientation		
Heterosexual	1308	92
Gay	16	1.13
Lesbian	13	1.00
Bisexual	53	4.00
Other	26	1.84
Household Income		
$29,000 or less	328	47
$30,000–$59,000	205	29
$60,000–$84,999	71	10
$85,000–$99,000	33	5
$100,000 and above	67	10
Education Attainment		
Less than high School	134	9
High School	399	28
Some College	616	43
Bachelor’s Degree or higher	288	20
HIV Testing		
Yes	238	66
No	455	34

**Table 2:** Bivariate correlations of study continuous variables on HIV stigma (N = 1437).

HIV Stigma	1						
HIV communication	0.1669 ***	1					
Bringing up the topic of HIV	−0.2139 ***	0.2413 ***	1				
HIV Communication with Partner	−0.1742 ***	0.4588 ***	0.2343 ***	1			
Perceived Stigma	−0.0214	0.1252 ***	0.122 ***	0.0998	1		
HIV Knowledge	0.0976	−0.0212	0.0278	0.0873 *	0.0575	1	
Age	−0.1376 ***	0.0072	0.047	0.139 ***	−0.0113	0.0519 *	1

*p* < 0.05 *, *p* < 0.01 **, *p* < 0.001 ***

**Table 3:** Sample Characteristics compared by HIV Testing (N = 1437).

		HIV Testing		
	Total	Yes	No	*χ* ^2^
Variables				
Race and Ethnicity				109.105 ***
African American or Black	271	123	144	
Hispanic	322	85	233	
Other, Non-Hispanic	57	13	43	
Two+ Races, Non-Hispanic	49	17	32	
White	728	107	621	
Gender				39.23 ***
Male	682	115	567	
Female	739	230	509	
Sexual Orientation				14.65 **
Heterosexual	1308	302	994	
Gay	16	4	12	
Lesbian	13	6	7	
Bisexual	53	23	30	
Other	26	19	7	
Condoms in Relationships				24.28 ***
Never	253	73	180	
Some of the time	134	55	79	
Most of the time	109	58	51	
All of the time	251	124	127	
Education Attainment				11.78 **
Less than high School	133	38	95	
High School	397	28	295	
Some College	608	43	450	
Bachelor’s Degree or higher	283	47	236	

*p* < 0.01 **, *p* < 0.001 ***.

**Table 4:** Multiple Linear Regression on HIV stigma (N = 1421).

HIV Stigma	B	SE	*p* > *t*	Beta
HIV Communication	0.02	0.03	0.65	0.02
Bringing up the topic of HIV	−0.13	0.04	0.001	-0.13
HIV Communication with Partner	0.10	0.04	0.004	0.12
Perceived Stigma	0.08	0.04	0.058	0.07
HIV Knowledge	−0.24	0.06	0.001	−0.14
Condoms in Relationship	−0.02	0.02	0.312	−0.04
Age	0.04	0.01	0.001	0.12
Gender (female reference)	0.06	0.06	0.275	0.04
Household income	0.01	0.01	0.014	0.09
Race and Ethnicity				
Black, Non−Hispanic	0.12	0.08	0.139	0.06
Other, Non−Hispanic	−0.09	0.14	0.531	−0.02
Hispanic	0.07	0.07	0.336	0.04
2+ Races, Non-Hispanic	0.24	0.15	0.123	0.05
Sexual Orientation				
Gay	0.82	0.22	0.001	0.13
Lesbian	0.37	0.24	0.118	0.05
Bisexual	0.46	0.13	0.001	0.12
Other, please specify	0.47	0.25	0.063	0.07

*p* < 0.01 **, *p* < 0.001 ***.

**Table 5:** Logistic Regression on HIV testing (N = 1421).

HIV Testing	OR	SE	*p* > *z*	95%
HIV communication	1.68	0.19	0.001	1.35, 2.09
Bringing up the topic of HIV	0.79	0.10	0.05	0.62, 1.00
HIV communication with partner	1.43	0.15	0.001	1.16, 1.76
Perceived Stigma	0.97	0.13	0.82	0.75, 1.26
HIV Knowledge	1.05	0.20	0.81	0.71, 1.54
Condoms in Relationship	1.32	0.10	0.001	1.14, 1.53
Age	1.21	0.05	0.001	1.12, 1.31
Gender (female reference)	1.49	0.28	0.04	1.03, 2.17
Household income	0.94	0.02	0.001	0.90, 0.97
Race and Ethnicity				
Black, Non-Hispanic	2.32	0.57	0.001	1.43, 3.77
Other, Non-Hispanic	1.06	0.48	0.89	0.44, 2.59
Hispanic	1.62	0.37	0.03	1.04, 2.53
2+ Races, Non-Hispanic	2.73	1.35	0.04	1.04, 7.19
Sexual Orientation				
Gay	0.38	0.27	0.17	0.10, 1.52
Lesbian	0.44	0.34	0.28	0.10, 1.95
Bisexual	1.42	0.60	0.41	0.62, 3.24
Other, please specify	0.97	0.73	0.97	0.23, 4.19

## Data Availability

The data is not available to the public without the consent of the PI.
